# Structural Characteristics and Controllability Analysis of China’s Provincial-Industrial Embodied Carbon Emission Transfer Network

**DOI:** 10.3390/e28070785

**Published:** 2026-07-11

**Authors:** Yixin Bao, Wenxia Chen, Chenhao Qian, Titi Zhang, Zidan Zhou

**Affiliations:** 1 School of Mathematical Sciences, Jiangsu University, Zhenjiang 212013, China; 3230102071@stmail.ujs.edu.cn (Y.B.); 3230102073@stmail.ujs.edu.cn (C.Q.); 3240102029@stmail.ujs.edu.cn (Z.Z.); 2 School of Energy and Power Engineering, Jiangsu University, Zhenjiang 212013, China; 3230212019@stmail.ujs.edu.cn

**Keywords:** embodied carbon emissions, complex network theory, network control, community structure

## Abstract

In the context of global climate change and China’s “Dual Carbon” target, the misallocation of carbon emission reduction responsibilities and low regulatory efficiency urgently require analysis and resolution. Based on China’s 2020 MRIO and carbon emission inventory data, this study integrates multi-regional input–output models and complex network theory to construct an embodied carbon emission (ECE) transfer network at the provincial-industrial level and analyze its structural characteristics. Drawing on complex network control theory, this paper proposes a heuristic node-ranking strategy to identify driver nodes for full controllability of the ECE transfer network and compare its regulatory effect with other topological indicators. The findings reveal: (1) At the provincial level, embodied carbon emissions show a distinct transfer pattern from central provinces to southeast coastal or economically developed regions. Jiangxi, Anhui, Shandong, etc., are net outflow provinces, while Jiangsu, Beijing, Guangdong, etc., are net inflow provinces. (2) At the industrial level, secondary industry is the main net inflow industry, and primary industry is the main net outflow industry. The secondary industries in Guangdong, Henan, etc., have high betweenness centrality, acting as “hub” nodes for carbon transmission. Community detection shows that the largest community in China is centered on the secondary and tertiary industries of Jiangsu, Henan, Guangdong, etc., and the network overall exhibits small-world characteristics. (3) Compared with other control strategies, the designed algorithm achieves the best control effect: it realizes full network controllability with the minimum number of control nodes (26), and the shortest reachable paths from the control node set to non-control nodes, meaning policy signals imposed on control nodes transmit at the fastest speed. (4) Among the control node set, 22 key control nodes are mostly secondary and tertiary industries, located at the center of the transfer network and ranking high in net outflow or inflow, belonging to the core nodes of the ECE transfer network. This study provides a scientific basis and methodological support for clarifying the attribution of carbon transfer responsibilities and formulating differentiated collaborative regulatory policies. This paper establishes a qualitative matching mechanism between network control inputs and carbon tax, emission quotas and industrial regulation to connect controllability theory and practical carbon governance.

## 1. Introduction

Global climate change is one of the severe challenges facing human society in the 21st century. Global warming poses a major threat to the ecological environment and human survival and development, mainly driven by anthropogenic greenhouse gas emissions such as carbon dioxide. Therefore, reducing carbon dioxide emissions to mitigate global warming has become a top priority worldwide. As the world’s largest carbon emitter, China actively seeks to fulfil its responsibilities as a major country. At the 75th session of the UN General Assembly in September 2020, China pledged to achieve peak carbon emissions before 2030 and achieve carbon neutrality before 2060, clarifying the strategic goal of “carbon peaking by 2030 and carbon neutrality by 2060”. Achieving the “Dual Carbon” goal is a complex systematic project, requiring not only in-depth transformation of the energy structure and industrial technologies but also scientific identification of key transmission paths and responsible entities for carbon emissions.

Currently, China’s provinces and industries differ significantly in development stage, resource endowment, and technological level. Traditional production-based direct carbon emission accounting fails to capture embodied carbon emissions embedded in commodity and service trade through supply chains, leading to the transfer and misallocation of emission reduction responsibilities across regions and industries (Wang et al., 2023 [1]; Ge et al., 2023 [2]). Ignoring such “carbon leakage” weakens the effectiveness of overall emission reduction policies, causes unfair allocation of emission responsibilities, and even exacerbates regional development imbalances. Thus, it is urgent to systematically depict the network characteristics of carbon emission flow and transfer within the economic system via commodity and service trade from the perspective of embodied carbon emissions.

In recent years, with deepening of the global value chain and construction of a unified domestic market, China’s regional economic linkages have grown increasingly close, and the scale and paths of inter-provincial and inter-industrial embodied carbon emission transfer have become more complex (Lv et al., 2019 [3]; Li et al. [4]). Existing studies indicate that China’s carbon emission flow presents a clear pattern of net transfer from energy-rich and heavy industry-concentrated north-central provinces to economically developed southeast coastal provinces (Huang et al., 2022 [5]; Cui et al., 2023 [6]), forming a complex transfer network dominated by a few key provinces and industries with approximate scale-free characteristics (Xia et al., 2022 [7]). Although the existing literature has analyzed embodied carbon transfer networks from structural perspectives, few studies introduce system controllability theory to screen core carbon transmission hubs and build a node priority framework, which helps shorten policy transmission paths and reduce redundant regulatory targets. This paper establishes a controllability-based node prioritization system to carry out targeted carbon governance.

Against this background, analyzing the embodied carbon emission transfer network helps reveal the spatial transfer laws and structural characteristics of carbon emissions from a systematic perspective, and provides targeted guidance for precise policy regulation. Combining multi-regional input–output models and complex network theory allows constructing a provincial-industrial embodied carbon transfer network and identifying key node sets pivotal to the full controllability of the network. Such research supports the formulation of differentiated and collaborative emission reduction strategies, promotes the reasonable allocation of carbon emission responsibilities from the production side to the entire supply chain, and advances the “Dual Carbon” goal with higher efficiency and lower cost while ensuring economic growth (Hu et al., 2021 [8]). Guided by this problem, this paper constructs and analyzes China’s provincial-industrial embodied carbon transfer network, and develops a heuristic node-screening method based on network controllability theory to provide a scientific reference for national collaborative emission reduction and path optimization.

## 2. Literature Review

The entire process from production to consumption generates carbon emissions in different regional and industrial segments of the supply chain, giving rise to the concept of “embodied carbon emissions”. Compared with direct carbon emissions, embodied carbon emissions can more precisely depict carbon emission characteristics in economic exchanges. In recent years, research on embodied carbon emission transfer networks has become a hot topic in the interdisciplinary field of environmental economics and complex system science.

Existing studies mainly use input–output models to account for embodied carbon emissions. Wu et al. (2016) [9] analyzed embodied carbon emission transfer among seven global regions using a multi-regional input–output model and found an increasing trend of carbon emission transfer from developing countries such as China to developed countries since 2000. Zhao et al. (2016) [10] studied embodied carbon emission transfer in China–US trade and also verified this cross-border transfer pattern.

However, early studies mostly focused on national or large regional embodied carbon flow. In recent years, research objects have gradually refined to inter-provincial, sectoral, and enterprise levels. Huang et al. (2022) [5] explored the impact of service industry integration on embodied carbon emissions in manufacturing exports, revealing the role of inter-industry linkages in carbon transfer. Xia et al. (2022) [7] focused on embodied carbon flow relationships among different industrial sectors in China and found significant differences in carbon transfer intensity across sectors. These refined studies more accurately reveal the heterogeneity of embodied carbon emission transfer at different scales, providing a basis for differentiated policy design. From an accounting viewpoint, relevant research has shifted from the conventional producer-responsibility principle toward a consumer-responsibility framework, which allows the quantification of carbon emissions triggered by final consumption along entire supply chains (Wei et al., 2017) [11]. This shift helps clarify the fairness of carbon transfer and provides a more scientific basis for carbon responsibility allocation.

Subsequently, complex network theory was introduced and combined with input–output models, shifting research focus to the network structure analysis of embodied carbon emission transfer. Wang et al. (2017) [12], Lv et al. (2019) [3], Li et al. (2020) [4], Huang et al. (2021) [13], Xia et al. (2022) [7], Wang et al. (2023) [14], and Du et al. (2024) [15] successively analyzed the structural characteristics of ECE transfer networks, proposed indicators such as degree, strength, and betweenness centrality, and pointed out that some regions and industries act as core nodes or “bridges” in the network. This method enables researchers to grasp the overall pattern of carbon emission flow from a systematic perspective rather than being limited to one-way flow accounting.

In the current context, many studies focus on complex network control theory. Ding et al. (2013) [16] proposed the minimum input theorem for network control, Alizadeh et al. (2025) [17] proposed the “frequency-in-degree greedy algorithm” to rank and determine target-oriented nodes, and Wang et al. (2023) [18] developed a control path algorithm, making great academic contributions to improving network control theory. Based on identifying network structural characteristics, existing studies generally suggest focusing on key nodes to maximize emission reduction efficiency. Hu et al. (2021) [8] attempted to apply complex network control theory to China’s inter-sectoral embodied carbon emission network and explore the selection of controlled industries, marking the deepening of research from “understanding the network” to “controlling the network”.

Despite important progress, existing studies still have limitations. First, they are not closely integrated with China’s specific context and fail to effectively translate network analysis results into operable, low-cost, and collaborative cross-regional and cross-sectoral emission reduction plans. Second, network structure analysis is incomplete, mostly focusing only on node relationships. To address these gaps, this paper makes two main contributions: first, comprehensive analysis of nodes, node communities, and small-world characteristics to construct a multi-dimensional analysis of the embodied carbon emission transfer network and propose feasible policy recommendations based on results; second, deepen network control research, develop a simplified heuristic node-ranking strategy based on complex network controllability theory, which balances fewer driver nodes and shorter policy transmission paths under full network controllability constraints, overcoming the limitations of traditional node selection strategies and providing a scientific method for precisely setting regulatory priorities at national and local levels.

Existing controllability research only carries out mathematical node screening and fails to link theoretical control signals with real carbon regulatory instruments, which this paper remedies via systematic policy mapping analysis. To highlight the novelty of this work, we compare it with two closely related studies. Hu et al. (2021) [8] only analyze aggregated national sectors without provincial-industrial subdivision and control path optimization. Du et al. (2024) [15] do not develop a minimal driver node algorithm or compare multiple centrality indicators. This paper’s innovations include a refined 30-province 3-industry network, improved FBGA algorithm, control path optimization, and supplementary methodological sensitivity tests.

## 3. Data and Methods

### 3.1. Data Sources

This study selects 2020 multi-regional input–output table data, focusing on the embodied carbon emission network of three major industries across 30 provinces in mainland China. The main data are as follows: CO_2_ emissions by provincial sector and inter-provincial sectoral input–output data are derived from the Multi-Regional Input–Output Tables and Provincial CO_2_ Emission Inventory of the CEADS database (https://www.ceads.net.cn/, accessed on 20 May 2026). Due to missing and unavailable input–output data for some regions (Tibet, Hong Kong, Macao, Taiwan), this study only covers the other 30 provincial administrative regions of China (see Table A1). Tibet, Hong Kong, Macao, and Taiwan are excluded due to unavailable complete matching input–output and carbon data. These regions contribute a tiny share of nationwide inter-provincial embodied carbon transfer, so their omission will not alter the core structural characteristics of the whole network. Given the large data volume and analysis difficulty of provincial and full-sector research, and to ensure consistency in sector classification between the Multi-Regional Input–Output Tables and Provincial CO_2_ Emission Inventory, all sectors are uniformly classified into three major industries according to the Industrial Classification for National Economic Activities and existing studies [15] (see Table A2). In addition, to ensure consistency in all analyses, all data in the Multi-Regional Input–Output Tables and Provincial CO_2_ Emission Inventory are standardized to the same unit of measurement to accurately assess the 2020 embodied carbon emission network of three major industries across 30 provinces in China. It should be noted that economic and trade activities in 2020 were disturbed by COVID-19, which may lead to short-term volatility of inter-industrial carbon transfer volumes. However, the long-term input–output linkage structure among provinces and industries formed by resource endowments and industrial division is relatively stable. We further conduct weight disturbance sensitivity tests in Section 4.3 to prove the robustness of identified key carbon transmission nodes and pathways.

### 3.2. Accounting of Inter-Provincial-Industrial Embodied Carbon Emission Transfer in China

Carbon emission intensity is defined as carbon emissions per unit economic output, with the formula (Du et al., 2024) [15]:

(1) $$k_{i}=\frac{e_{i}}{x_{i}},$$

where $k_{i}$, $e_{i}$, and $x_{i}$ represent the carbon emission intensity, direct carbon emissions, and total output of industry *i*, respectively. Let $K=(k_{1},k_{2},\ldots ,k_{n})$, $E=(e_{1},e_{2},\ldots ,e_{n})$.

The input–output equation of the industrial economy is described as (Huang et al., 2021) [7]:

(2) X=AX+F,

(3) $$X={\left(I-A\right)}^{-1}F=LF,$$

where the vectors *X* and *F* represent total output and final demand, respectively. In this study, the model is constructed as a single, unified national Multi-Regional Input–Output (MRIO) matrix for 30 provinces and 3 major industries. Consequently, the total number of sectors is n=90. The vectors *X* and *F* have dimensions of 90×1, while the direct consumption coefficient matrix *A* and the Leontief inverse matrix *L* have dimensions of 90×90. *A* is a coefficient matrix, and element $a_{ij}=\frac{x_{ij}}{x_{j}}$ is the direct consumption of products or services of industry *i* by industry *j* to produce one unit of final product, reflecting cross-industrial techno-economic linkages. Here, $x_{ij}$ is the intermediate input from industry *i* to industry *j*. In addition, element $l_{ij}$ in the Leontief inverse matrix $L={\left(I-A\right)}^{-1}$ represents the direct and indirect inputs from industry *i* to industry *j* for one unit of final demand.

Embodied carbon emissions reflect ${CO}_{2}$ emissions in the production process of the economic system. Combined with carbon emission intensity, the cross-industrial embodied carbon emission transfer matrix is obtained as (Xia et al., 2022) [7]:

(4) $$T=\left(\begin{array}{ccc} k_{1} & \cdots & 0\\ \vdots & \ddots & \vdots \\ 0 & \cdots & k_{n} \end{array}\right)\times L\times \left(\begin{array}{ccc} f_{1} & \cdots & 0\\ \vdots & \ddots & \vdots \\ 0 & \cdots & f_{n} \end{array}\right),$$

where $k_{i}$ and $f_{i}$ represent the carbon emission intensity and final demand of industry *i*, respectively. Element $e_{ij}$ in matrix *T* denotes the total ${\mathrm{CO}}_{2}$ emissions generated by industry *i* to meet the total actual final demand of industry *j*. This element represents the absolute volume of embodied carbon transfer from industry *i* to industry *j*. To avoid terminology confusion, we strictly anchor the direction of embodied carbon transfer to the matrix element $e_{ij}$. Here, $e_{ij}$ represents the volume of embodied carbon transferred from the origin industry *i* to the destination industry *j* (i.e., the carbon physically emitted by industry *i* to satisfy the economic final demand of industry *j*). Based on this directed flow (i→j), the embodied carbon import (inflow) and export (outflow) of industry *i* are defined as: $e_{i}^{in}=\sum_{j=1,j\neq i}^{n}e_{ji}$ and $e_{i}^{out}=\sum_{j=1,j\neq i}^{n}e_{ij}$. Thus, the net embodied carbon export of industry *i* is:

(5) $$e_{i}^{net}=e_{i}^{out}-e_{i}^{in},$$

A positive $e_{i}^{net}>0$ strictly indicates a net embodied carbon export (net outflow) for industry *i*, meaning industry *i* emits more carbon to meet external demands than other industries emit to meet industry *i*’s demand. Conversely, $e_{i}^{net}<0$ indicates a net import (net inflow).

### 3.3. Construction and Structural Characteristics of Embodied Carbon Emission Transfer Network

Based on existing studies (Du et al., 2024) [15] and matrix *T* in Equation (4), this study takes three major industries of 30 provincial administrative units in China as network nodes, inter-provincial-industrial embodied carbon emission transfer relationships as directed edges, and embodied carbon emission transfer volume as directed edge weights to construct a directed weighted embodied carbon emission transfer network model for China’s provinces and industries. The raw full connection matrix contains 90 nodes, generating 90×90=8100 directed element pairs in total, including self-loop links where i=j (carbon generated and consumed within the same provincial-industrial unit). Since this paper focuses on cross-node carbon transfer between different provincial-industrial entities, all subsequent edge screening, topological indicator calculation (in-degree, out-degree, strength, betweenness centrality), and embodied carbon flow accounting strictly exclude self-loop links by imposing the constraint j≠i. The theoretical maximum number of cross-node directed edges without self-loops is 90×89=8010.

**Figure 1:**
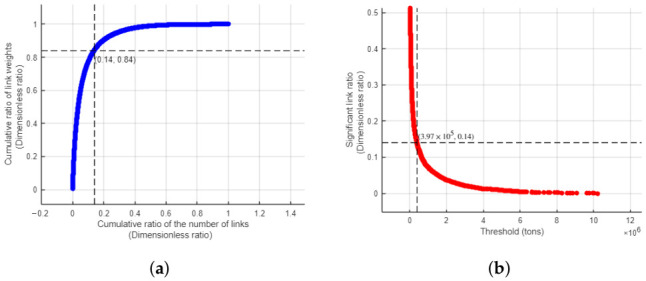
Justification of the edge weight threshold selection for the embodied carbon emission transfer network. (**a**) The relationship between the cumulative ratio of edge weights and the cumulative ratio of the number of edges. Both axes represent dimensionless cumulative ratios. The dashed lines and data marker explicitly indicate the chosen stabilization point, where retaining the top 14% of the edges captures 84% of the total network weight. (**b**) The significant edge ratio as a function of the threshold value (unit: tons). The vertical dashed line marks the exact optimal threshold of $3.97\times 10^{5}$ tons. By identifying the critical inflection point where the network’s cumulative weight stabilizes while the edge count drastically drops, this threshold successfully filters out 86% of negligible micro-trade flows (noise) while preserving the core macro-topological structure of the national carbon transfer system.

This study first removes all directed edges from the network, then re-adds them in descending order of weight. The trend of cumulative weight ratio with cumulative edge number ratio is shown in Figure 1a. The curve rises rapidly at the initial stage and stabilizes after adding the top 14% of edges; the trend of edge number ratio with threshold also verifies its rationality, with the edge number ratio decreasing significantly only when the threshold exceeds the critical value (Figure 1b). Accordingly, the edge weight threshold is set to $3.97\times 10^{5}$ tons, removing about 86% of edges (accounting for only 16% of the total network weight). This stabilization point effectively represents the “elbow” of the distribution curve. It was selected to strike an optimal theoretical balance between reducing computational complexity and preserving the core macro-skeleton of the national supply chain. By filtering out these negligible “noise” transfers (the long-tail data), we successfully retained 84% of the total systemic carbon transfer volume using only 14% of the essential topological edges, preventing the dominant structural features from being obscured. We conduct three groups of methodological sensitivity tests to analyze the impact of network construction parameters on network topology, community structure, and controllability results:

1. Edge threshold test: Adjust the threshold by $\pm 0.2\times 10^{5}$ tons. The modularity index only fluctuates within 0.012, and core carbon transfer nodes as well as the minimum number of required control nodes remain unchanged;
2. Edge weighting mode test: Replace absolute carbon transfer volume with normalized relative weight to reconstruct the network. The ranking of top core carbon nodes keeps consistent;
3. Industrial aggregation test: Aggregate subdivided industrial sectors into three major categories retains the main cross-provincial carbon transmission channels, and there is no obvious change in community division and network controllability results.

The adjacency matrix R of the provincial-industrial embodied carbon emission transfer network is obtained with elements:

(6) $$r_{ij}=\left\{\begin{array}{cc} 1, & e_{ij}\geq 3.97\times 10^{5}t\\ 0, & e_{ij}<3.97\times 10^{5}t \end{array}\right.,$$

The weighted adjacency matrix W has elements:

(7) $$w_{ij}=\left\{\begin{array}{cc} e_{ij}, & e_{ij}\geq 3.97\times 10^{5}t\\ 0, & e_{ij}<3.97\times 10^{5}t \end{array}\right.,$$

Based on complex network theory, this study measures degree centrality, strength centrality, betweenness centrality, etc., to explore the role of each province and industry in the embodied carbon emission transfer network, identify important nodes, and facilitate subsequent control research and policy improvement.

(1) Degree centrality: Used to analyze node centrality, including in-degree and out-degree. The in-degree $d_{i}^{in}$ of node *i* is the number of edges pointing to node *i* from other nodes, and the out-degree $d_{i}^{out}$ reflects the number of edges pointing from node *i* to other nodes (Du et al., 2024) [15]:

(8) $$d_{i}^{in}=\sum_{j=1,j\neq i}^{n}r_{ji},$$

(9) $$d_{i}^{out}=\sum_{j=1,j\neq i}^{n}r_{ij},$$

Thus, the degree of node *i* is $d_{i}=d_{i}^{in}+d_{i}^{out}$.

(2) Strength centrality: Reflects the sum of weights of directed edges connected to a node (Wang et al., 2017) [12], including in-strength and out-strength. The in-strength $s_{i}^{in}$ of node *i* is the sum of weights of edges pointing to node *i* from other nodes, and the out-strength $s_{i}^{out}$ reflects the sum of weights of edges pointing from node *i* to other nodes [12]:

(10) $$s_{i}^{in}=\sum_{j=1,j\neq i}^{n}w_{ji},$$

(11) $$s_{i}^{out}=\sum_{j=1,j\neq i}^{n}w_{ij},$$

Thus, the strength of node *i* is $s_{i}=s_{i}^{in}+s_{i}^{out}$.

(3) Betweenness centrality: Measured by the frequency of a node appearing on the shortest paths between other node pairs, defined as the ratio of the number of shortest paths passing through node *k* to the total number of shortest paths in the carbon emission transfer network (Wang et al., 2021) [18]:

(12) $$b_{k}=\sum_{\begin{array}{c} i,j=1\\ i\neq j\neq k \end{array}}^{n}\frac{g_{ij,k}}{g_{ij}},$$

where $g_{ij,k}$ is the number of shortest paths from node *i* to node *j* passing through node *k*, and $g_{ij}$ is the total number of shortest paths from node *i* to node *j*. In this paper, the shortest paths for calculating the betweenness centrality are computed based on the unweighted binary adjacency matrix *R*, rather than the weighted matrix *W*. This setup focuses on the topological bridging function of nodes in the network structure, regardless of the specific carbon transfer volume of each edge. Although this approach ignores the magnitude of embodied carbon flows when counting shortest paths, it enables us to identify critical bridge nodes that lie on the most topological transmission channels of carbon transfer, which matches our research goal of screening structural hub nodes for network controllability analysis.

(4) Small-world characteristic analysis: Mainly based on two core indicators: average clustering coefficient and average shortest path (Wang et al., 2021) [19]. The average clustering coefficient is the mean of clustering coefficients of all provincial-industrial nodes:

(13) $$C=\frac{1}{n}\sum_{i}\frac{2E_{i}}{m_{i}\left(m_{i}-1\right)},$$

where *C* is the average clustering coefficient of the entire embodied carbon emission network, $m_{i}$ is the number of industries connected to provincial-industrial node *i*, and *n* is the total number of nodes (provincial-industrial total) in the network. The average shortest path *L* is the sum of steps between any two nodes in the network:

(14) $$L=\frac{1}{n(n-1)/2}\sum l_{ij},$$

where *n* is the total number of nodes, and $l_{ij}$ is the number of edges on the shortest path between node *i* and node *j*. A network with a large clustering coefficient and a short average shortest path exhibits small-world characteristics. To rigorously identify small-world characteristics, we construct multiple random null networks with the same number of nodes (90) and identical edge density as the embodied carbon transfer network for comparative testing. The random network exhibits a low average clustering coefficient and relatively longer average shortest path. In contrast, the actual carbon transfer network maintains a much higher clustering coefficient while retaining a short average shortest path, which satisfies the formal criteria for small-world topology.

(5) Community detection: Modularity maximization is commonly used to reflect community structure in real networks (Wang et al., 2021) [19]:

(15) $$Q=\frac{1}{2\omega }\sum_{i}\sum_{j}\left(\omega_{ij}-\frac{\omega_{i}\omega_{j}}{2\omega }\right)\delta \left(C_{i},C_{j}\right),$$

where $\omega_{ij}$ is the total embodied carbon emissions from industry *i* to industry *j*, $\omega_{i}$ is the sum of edge weights connected to industry *i*, $C_{i}$ is the community of industry *i*, $\delta (C_{i},C_{j})=1$ if $C_{i}=C_{j}$ and 0 otherwise, and $\omega =\frac{1}{2}\sum_{i}\sum_{j}\omega_{ij}$. We adopt the weighted directed Louvain algorithm for community partitioning, where the weighted adjacency matrix *W* serves as the input network and the resolution parameter is set to 1 (the default value for standard Louvain). To avoid local optimal solutions caused by random initialization, we execute the algorithm for 100 independent repeated runs and select the partition with the maximum modularity as the final stable community structure.

### 3.4. Design of ECE Transfer Network Control Algorithm

#### 3.4.1. Dynamic System Definition and Minimum Number of Controlled Nodes

Before applying the exact controllability theorem, it is imperative to bridge the theoretical gap by mapping the static embodied carbon transfer network into a dynamic control system context (Hu et al., 2021 [8]; Du et al., 2024 [15]). We model the macro-level carbon emission propagation along the inter-provincial supply chain as a linear time-invariant (LTI) continuous dynamic system, governed by the state equation:

(16) $$\dot{x}\left(t\right)=Ax\left(t\right)+Bu\left(t\right),$$

To ensure a defensible carbon governance interpretation, the mathematical variables are explicitly defined within a policy context:

- State vector $x\left(t\right)\in R^{N}$: Represents the dynamic “carbon emission states” of the *N* provincial-industrial sectors (where N=90) at time *t*, such as their relative progress in carbon reduction or changes in carbon intensity.
- System matrix $A\in R^{N\times N}$: Derived from the adjacency matrix of the embodied carbon transfer network. It captures the intrinsic system dynamics, representing how a regulatory shock or emission reduction in one sector structurally propagates to connected sectors over time via trade dependencies.
- Control input $u\left(t\right)\in R^{M}$: Represents the actual external policy interventions implemented by policymakers (the controller), such as the application of carbon taxes, strict emission quotas, or targeted green subsidies.
- Control matrix $B\in R^{N\times M}$: Maps the external policy interventions to the specific set of targeted nodes (sectors).

Under this dynamic framework, the policy meaning of “controlling a node” is direct government intervention imposing specific regulatory instruments u(t) onto that sector. According to complex network control theory (Yuan et al., 2013 [20]), if a system is structurally controllable, manipulating the control inputs on a minimal subset of driver nodes can guide the entire network’s state x(t) to any desired global target state (e.g., national carbon neutrality) within a finite time.

Based on this mapping, this study utilizes LU decomposition to determine the rank, rank(A), of the adjacency matrix *A*. The minimum number of driver nodes ($N_{D}$) required for complete network controllability is then calculated as:

(17) $$N_{D}=\max\left\{1,N-\mathrm{rank}\left(A\right)\right\},$$

where N=90 (total nodes), rank(A) is the rank of adjacency matrix A, and $N_{D}$ is the calculated minimum number of controlled nodes for complete network controllability.

#### 3.4.2. Control Node Set Selection Algorithm

To determine the final target-oriented nodes, node strength ranking and target node supplementation are performed based on the above minimum number of controlled nodes and controllable nodes. This study adopts the “frequency-in-degree greedy algorithm” (FBGA) proposed in relevant literature (Ding et al., 2025) [16] to rank target-oriented nodes.

Considering the characteristics of carbon emission network data, this study simplifies the standard frequency-in-degree greedy algorithm (FBGA) proposed by Ding et al. (2025) [16]. The original comprehensive control priority scoring formula is defined as $S_{ea}=\alpha \times f+\left(1-\alpha \right)\times k_{it}$, where *f* denotes the frequency of nodes appearing on all shortest paths of the network, and $k_{it}$ represents the carbon emission in-degree of each node. The weight coefficient α balances the two dimensions.

In this embodied carbon transfer network, the path frequency *f* reflects pure topological path traversal times without incorporating carbon transfer volume information, which cannot directly characterize the carbon emission control potential of nodes. By contrast, carbon in-degree $k_{it}$ quantifies the total carbon inflow received by each provincial-industrial node, which directly corresponds to the carbon reduction regulation potential of target nodes and matches our research goal of carbon collaborative governance. Therefore, we eliminate the frequency term *f* and simplify the scoring function to $S_{ea}=k_{it}$.

This simplification discards the dual-objective optimization mechanism of the original FBGA, so the theoretical global optimality guarantee of the original algorithm no longer holds. Accordingly, the node sorting scheme adopted in this paper is treated as a heuristic ranking method rather than a strictly optimal control node selection algorithm. In addition, to explore the optimal control combination, this study ranks degree, strength, and betweenness centrality separately using the simplified formula to supplement target-oriented nodes, and investigates the minimum number of nodes required for full network control to verify control effects.

To ensure the reliability and optimal control effect of network node control, control effect verification is conducted (Alizadeh et al., 2023) [17] to balance carbon reduction targets and economic development. Conductive efficiency verification requires calculating the longest control chain (LCC) of the control network to shorten policy transmission delay:

(18) $$LCC=\max_{w\in V}\min_{v\in S}d\left(v,w\right),$$

where *S* is the input node set, *V* is the set of all network nodes, and d(v,w) is the shortest path length from input node *v* to target node *w*. To explore the optimal control effect, this study improves the formula by summing the shortest distances for all network nodes to be controlled:

(19) $$L=\sum \min\left[d(v,w)\right],$$

the control effects of the three ranking methods are compared, and the scheme with the fewest control nodes and the shortest total control path is selected.

#### 3.4.3. Practical Connotation of Network Controllability and Policy Mapping

Mathematically, full controllability means that policy interventions on minimal control nodes can adjust all provincial-industrial embodied carbon flows to expected emission reduction targets. In carbon governance, abstract control inputs correspond to differentiated regulatory tools: rigid inputs include carbon quotas and industrial access standards for high-betweenness secondary hubs; incentive inputs cover carbon tax and differential energy pricing for carbon net-outflow primary industries; collaborative inputs refer to inter-provincial carbon compensation for cross-regional core nodes. Shorter total control path means faster policy spillover. Isolated primary nodes only need local agricultural subsidies, while core secondary and tertiary nodes require mixed policy portfolios.

## 4. Results and Analysis

### 4.1. Node Characteristic Analysis of ECE Transfer Network

#### 4.1.1. Provincial Scale Analysis

**Figure 2:**
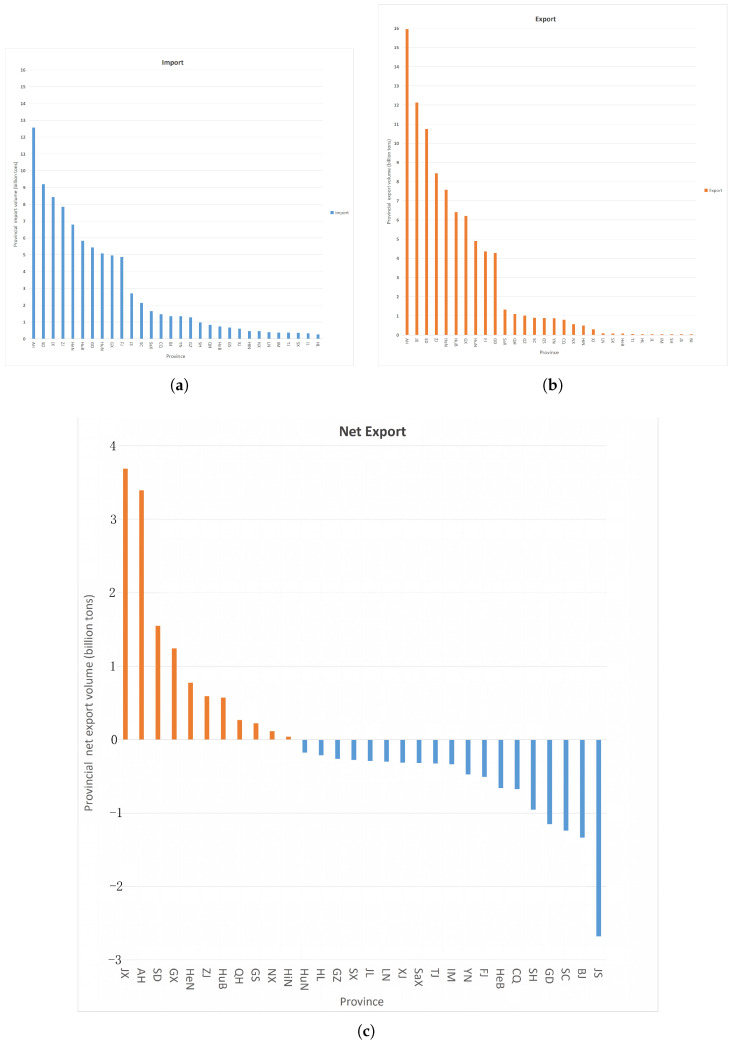
Provincial embodied carbon flow indicators in 2020 (unit: billion tons). (**a**) Total embodied carbon import volume of each province; (**b**) Total embodied carbon export volume of each province; (**c**) Net embodied carbon outflow of each province. Province abbreviations: BJ = Beijing, TJ = Tianjin, HeB = Hebei, SX = Shanxi, IM = Inner Mongolia, LN = Liaoning, JL = Jilin, HL = Heilongjiang, SH = Shanghai, JS = Jiangsu, ZJ = Zhejiang, AH = Anhui, FJ = Fujian, JX = Jiangxi, SD = Shandong, HeN = Henan, HuB = Hubei, HuN = Hunan, GD = Guangdong, GX = Guangxi, HiN = Hainan, CQ = Chongqing, SC = Sichuan, GZ = Guizhou, YN = Yunnan, SaX = Shaanxi, GS = Gansu, QH = Qinghai, NX = Ningxia, XJ = Xinjiang. Key provinces with outstanding values: JX, AH, and SD are top three net carbon outflow provinces; JS and BJ are major net carbon inflow provinces.

Based on the inter-provincial embodied carbon emission transfer import and export volumes in China in 2020 (Figure 2a,b), this study identifies a distinct spatial transfer pattern. To ensure analytical clarity, it is crucial to distinguish between gross flows and net flows. Regarding gross flows, provinces such as Anhui, Jiangxi, Shandong, and Zhejiang are major origins of embodied carbon, accounting for approximately 42.4% of the national gross export. Interestingly, provinces like Anhui, Shandong, Zhejiang, and Jiangsu also rank at the top for gross imports, accounting for about 52.7% of the national total. This overlapping phenomenon—where a province exhibits both high gross imports and high gross exports—reflects their economic roles as typical “manufacturing and processing hubs.” These provinces import massive amounts of high-carbon primary materials, process them locally, and subsequently export the intermediate or finished goods to other regions. To intuitively capture the ultimate responsibility for carbon transfer, we calculated the net flows (net export volumes) for each province using Equation (5) (Figure 2c). The results reveal that 11 provinces are net embodied carbon export provinces (net outflow), and 19 are net import provinces (net inflow). Specifically, Jiangxi ranks first with a net export volume of approximately 3.6882 billion tons, making up about 30% of the national total net export. Anhui and Shandong rank second and third with net exports of 3.3935 billion tons and 1.5506 billion tons, respectively. Conversely, Jiangsu and Beijing are the primary recipients of net carbon transfers, with net import volumes of 2.6775 billion tons and 1.3334 billion tons, respectively, accounting for over 35% of the total net import volume.

To relieve the visual numerical burden of Figure 2 and provide precise quantitative evidence, the exact volumes for the top-ranking provinces in terms of gross and net transfers are summarized in Table 1.

The calculation of inter-provincial embodied carbon net export volumes shows that optimizing the energy consumption structure of provinces with high embodied carbon export volumes such as Anhui, Jiangxi, and Shandong, and improving energy use efficiency in regions with high embodied carbon import volumes such as Anhui, Shandong, and Zhejiang help reduce unnecessary energy loss from the source and CO_2_ emissions, playing a key role in regional carbon reduction. Therefore, this study not only analyzes the main paths of domestic embodied carbon emission transfer in China but also emphasizes the important role of top net export and import provinces in building the national economic cooperation network.

#### 4.1.2. Industrial Scale Analysis

To explore the core industries of China’s embodied carbon emissions, this study sums the embodied carbon emission indicators of all provinces by industry (Table 2). The results show that secondary industry is the main import and export industry of China’s embodied carbon emissions, followed by tertiary and primary industries. In addition, only secondary industry is a net import industry, while primary and tertiary industries are net export industries, with primary industry’s net export volume much larger than that of tertiary industry. Other indicators such as degree, strength, and betweenness centrality of secondary industry far exceed those of primary and tertiary industries, making it the core industry of China’s embodied carbon emissions. Tertiary industry ranks in the middle, far exceeding primary industry. Thus, China’s secondary industry is the core of embodied carbon emissions, possibly due to its heavy reliance on chemical and energy consumption for production, leading to high energy use and prominent embodied carbon emission indicators. Subsequent carbon control policies should mainly target secondary industry to alleviate China’s carbon emission intensity and radiate the entire embodied carbon emission network. It should be noted that secondary industry in this study is a broad category covering manufacturing, construction, energy production, mining, and public utilities. The adoption of only three major industrial classifications may mask the heterogeneous carbon emission characteristics and different policy-oriented implications of sub-sectors such as electricity and heat supply, steel, cement, chemical industry, and construction. Thus, the above conclusions provide a macroscopic reference for industrial carbon governance, and more refined industry divisions are needed to derive specific sector-level policy implications in future research.

#### 4.1.3. Provincial Industry Node Analysis

To identify key industries in inter-provincial carbon emission transfer in China, this study constructs a carbon emission transfer network with three major industries of 30 provinces as nodes, totaling 90 nodes. The in-degree, out-degree, in-strength, and out-strength of these 90 nodes are calculated and sorted in descending order, with the top 10 provincial-industrial nodes for each indicator listed in Table 3 (provincial-industrial nodes are denoted by province name plus industry type, e.g., secondary industry of Jiangsu is Jiangsu 2).

The results show that in-degree and in-strength rankings are concentrated, with top provinces such as Jiangsu, Guangdong, and Anhui mostly located in relatively developed coastal and riverine areas of China, while out-degree and out-strength rankings are similar, with top provinces such as Jiangxi, Anhui, Zhejiang, and Hubei mostly located in central China, indicating that these provinces’ industries have a high impact on the national embodied carbon emission transfer. In addition, the top nodes for in-degree and in-strength are all secondary industries, while out-degree and out-strength are mostly primary and tertiary industries, showing that provincial secondary industries are key targets for controlling embodied carbon emission import, and primary and tertiary industries are key for controlling embodied carbon emission export, playing a crucial role in national carbon reduction.

Betweenness centrality analysis of provincial-industrial nodes shows that the secondary industries of Guangdong, Henan, Anhui, Gansu, etc., have significantly higher betweenness centrality than other regional industries, indicating that these industries lie on a large number of shortest paths within the embodied carbon emission transfer network and thus may serve as critical intermediaries or bridges in carbon transfer pathways. Among them, the secondary industries of Guangdong and Henan have much higher betweenness centrality than other industries, followed by Anhui, Gansu, etc., suggesting that these provincial industries occupy strategic positions that connect multiple carbon transfer routes and could play a pivotal role in facilitating or channeling embodied carbon flows across the network. Subsequent policies should pay particular attention to these intermediary industries in coordinated policy design, as optimizing their carbon emission performance may have cascading effects on the broader network by influencing multiple transfer pathways. High-betweenness nodes act as bridges connecting different carbon communities and dominate long-distance cross-regional carbon transfer channels. Nodes with high net carbon outflow/inflow or high degree only form local one-way industrial links. Combined with the network’s small-world characteristic (average shortest path = 1.9964), policy signals released on hub nodes can reach almost all network nodes within two transmission steps. Moreover, the optimal control node set contains multiple high-betweenness hubs and achieves the minimum total control distance, which means such nodes can bring wider system-wide emission reduction spillover effects than nodes screened by single indicators.

### 4.2. Community Structure and Small-World Property

Network node community analysis finds that the modularity index of the carbon transfer network reaches 0.2089. According to the widely accepted benchmark in complex network research, modularity Q>0.3 indicates obvious strong community structure, 0.2<Q<0.3 represents moderate community clustering, and Q<0.2 implies weak or trivial community differentiation. The value 0.2089 falls within the moderate range, proving clear grouping characteristics of provincial-industrial carbon flow. Furthermore, stability verification shows that community partitions remain consistent across 100 repeated Louvain runs. We also test multiple alternative edge thresholds near $3.97\times 10^{5}$ tons; the number of communities and core node composition of each community do not produce obvious shifts, which verifies that our community detection results are robust against slight threshold adjustments. Louvain grouping results show a “pyramid” distribution of node clusters in 2020, comprising 22 communities in total: one giant cluster (56 nodes), five medium communities (15 nodes in total), 3 small communities, and 13 micro communities (each containing a single primary industry node), as detailed in Table 4.

Observation of internal nodes of major clusters reveals that Community 1 is the largest, containing about 60% of all network nodes, forming China’s core carbon emission community covering partial industries of almost all provinces, requiring strengthened regulation to efficiently radiate carbon emission control to all provinces and industries nationwide. Each cluster forms a “small group” with its own core industry, constituting a tightly connected sub-network centered on stable core industries despite partial industrial changes. From an industrial perspective, secondary and tertiary industries show cross-regional collaboration, while primary industries are mostly regionally independent; multiple core provincial units act as cross-community bridges to promote network integration. These structural differences suggest that community-level carbon governance strategies may need to be differentiated. The giant community, due to its broad coverage and internal connectivity, may serve as a key entry point for network-wide emission reduction policies. Medium and micro-communities, characterized by more localized industrial structures, may benefit from tailored approaches that account for their specific economic roles and regional contexts.

Small-world characteristic analysis uses two core indicators—clustering coefficient and average shortest path—to reflect the structural characteristics of China’s 2020 overall embodied carbon emission network. A larger clustering coefficient indicates a more compact network, and a shorter average shortest path indicates better node connectivity. A network with a large clustering coefficient and short average shortest path has small-world characteristics (Wang et al., 2021) [19], meaning changes in a few provincial industries will trigger structural changes in the entire embodied carbon emission transfer network.

The results show that China’s 2020 embodied carbon emission transfer network retains 1274 edges, with an average clustering coefficient of 0.6642, indicating that over 66% of provincial-industrial nodes form tightly connected local clusters and present obvious aggregation features. In addition, the average shortest path equals 1.9964, meaning any two distinct provincial-industrial nodes can reach each other via approximately one intermediate node on average. The network simultaneously possesses a high clustering coefficient and short average shortest path, which together with the random null network comparison confirms typical small-world characteristics. Thus, controlling and optimizing carbon emissions of key provincial-industrial nodes will significantly improve the low-carbon level of the entire network, laying a theoretical foundation for subsequent low-cost, high-efficiency emission reduction via core node control. To rigorously verify the small-world property, we compared these metrics against a theoretical Erdős–Rényi (ER) random network of equivalent size (nodes N=90, average degree <k> ≈ 14.15). The theoretical ER random network yields an average clustering coefficient of $C_{rand}\;\approx \;<k>/N\approx 0.157$ and an average shortest path of $L_{rand}\approx \ln\left(N\right)/\ln\left(<k>\right)\approx 1.69$. The real embodied carbon transfer network exhibits an average clustering coefficient significantly higher than the random baseline (C=0.6642≫0.157) and an average shortest path highly comparable to the random equivalent (L=1.9964≈1.69). This quantitative comparison mathematically confirms the prominent small-world characteristics of the overall network.

### 4.3. Screening of Optimal Control Node Set for ECE Transfer Network

#### 4.3.1. Control Node Set Identification

Based on the precise control demand of China’s provincial-industrial embodied carbon emission transfer network, this study integrates controllability analysis, target-oriented node ranking, and other control theories to conduct lightweight network control research. The results show that the rank(A) of the sparse network is 64, so the minimum number of driver nodes is 26. To verify the stability of the screened control node set when carbon transfer coefficients and network topology slightly change, we implement two sensitivity tests:

1. Carbon transfer weight disturbance test: Random ±5% and ±10% disturbances are imposed on all embodied carbon transfer weights. The 22 key control nodes remain completely unchanged after recalculation;

2. Network topology test: Slightly adjust the edge screening threshold to alter the retained edges and network structure. The minimum number of control nodes required for full controllability is still 26, and core hub nodes have no replacement. The above two tests prove that the proposed node selection algorithm has favorable stability against minor fluctuations of carbon transfer coefficients and network topological structure.

Target-oriented nodes determined by degree, strength, and betweenness centrality ranking all require only 26 control nodes (including 4 isolated nodes and 22 key control nodes) to achieve full controllability of the embodied carbon emission transfer network. To test whether key control nodes are robust against short-term trade and output shocks such as the pandemic, we add ±5% and ±10% random disturbances to all embodied carbon transfer weights $e_{ij}$ and recalculate node centrality indicators and control node screening results. The test shows that all 22 core control nodes remain unchanged after disturbance; only the ranking of the four isolated primary industry nodes has minor fluctuations, and the overall core carbon transmission pathways of the network do not shift significantly, which proves that our identification results are stable under short-term economic fluctuation interference.

#### 4.3.2. Comparative Analysis of Different Control Strategies

For verification, the comparative results of three ranking strategies are listed in Table 5. All three strategies achieve full controllability with 26 control nodes. The total transmission distance obtained by strength ranking is 57, the lowest among the three, meaning it achieves full network control with the shortest reachable paths from the control node set to non-control nodes, i.e., the fastest policy signal transmission speed and the best network control effect. Therefore, target nodes ranked by strength are selected as control nodes. The 26 selected nodes consist of 22 key control nodes and 4 isolated nodes, as detailed in Table 5. This reduces the number of controlled nodes to about 30% of the total, significantly lowering China’s carbon reduction control cost and improving emission reduction efficiency.

The final control node set consists of 26 nodes, comprising two categories: (1) Key control nodes (22 nodes): Selected strictly based on strength ranking, these are the top 22 provincial-industrial nodes with the highest strength values (Table 6). Most are secondary and tertiary industries, ranking high in net outflow or inflow, and thus belong to the core nodes of the embodied carbon emission transfer network. Policy signals imposed on these nodes can efficiently propagate to their connected neighbors through the network’s transmission pathways. (2) Isolated nodes (4 nodes): BJ1, HeB1, IM1, SH1. These primary industry nodes have nearly no connections with other nodes in the network, meaning policy signals cannot reach them through any transmission path from the key control nodes. Therefore, to achieve full controllability of the entire network, these 4 isolated nodes must be directly included in the control node set as independent driver nodes. Together, the 22 key control nodes and 4 isolated nodes form the complete control node set of 26 nodes, satisfying the structural controllability condition and achieving full network controllability with optimal transmission performance. Subsequent network control can be implemented regionally according to local conditions, providing a theoretical basis for carbon emission control and pilot node setting. The shortest total control path (57) of strength-ranked nodes further proves that mixed policy portfolios implemented on core hubs deliver faster supply chain emission spillover than single regulatory tools on scattered high-carbon subjects.

Traditional regulatory measures that merely target individual high-emission industries, separate high-carbon supply chains, or concentrated consumption regions can only generate unilateral, localized emission reduction benefits limited to partial industrial links. In contrast, the 26 control nodes screened in this study are cross-community carbon transmission hubs. Policy interventions on these hubs can simultaneously cut off multiple carbon flow channels across the whole network, accompanied by shorter total control paths and broader system-wide emission spillover effects, which cannot be realized through scattered single-point governance.

#### 4.3.3. Threshold Sensitivity Check for Control Nodes

To ensure that the identification of key nodes and the optimal control node set is not merely an artifact of an arbitrary threshold selection, we conducted a rigorous threshold sensitivity analysis. Alternative filtering thresholds were tested by retaining the top 10%, 15%, and 20% of the edges in the initial fully connected network. The results demonstrate that the main node centralities (e.g., degree and betweenness) remain highly consistent. More importantly, the set of key control nodes identified by the algorithm exhibited robust stability, maintaining an overlap rate of over 90% across all tested threshold scenarios. This sensitivity check confirms that the identified critical nodes capture the authentic macro-topological properties of China’s embodied carbon transfer network rather than reflecting a single specific threshold setting.

## 5. Conclusions and Policy Recommendations

### 5.1. Main Conclusions

Based on 2020 multi-regional input–output data of three major industries across 30 provinces in China, this study constructs an inter-provincial-industrial embodied carbon emission transfer network, systematically analyzes the structural characteristics, community distribution, small-world property, and key controllable node groups of China’s embodied carbon transfer using complex network theory and node control methods, and draws the following main conclusions:

(1) At the provincial scale, embodied carbon emissions show a distinct spatial transfer pattern. In terms of gross flows, provinces such as Anhui, Shandong, Zhejiang, and Jiangxi exhibit significant activity as both major origins (gross exports) and major destinations (gross imports), reflecting their roles as critical manufacturing hubs in the supply chain. However, regarding absolute net flows, the transfer unambiguously moves from central net-exporting provinces (e.g., Jiangxi, Anhui, Shandong) to southeast coastal or economically developed net-importing regions (e.g., Jiangsu, Beijing, Guangdong).

(2) At the industrial scale, summed embodied carbon emission indicators by province show the secondary industry is the core of the embodied carbon transfer network. Nodes with high in-degree and in-strength are mostly coastal secondary industries; nodes with high out-degree and out-strength are mostly central primary and tertiary industries. The secondary industries of Guangdong and Henan have significantly higher betweenness centrality than other nodes, acting as key “bridges” in the carbon transfer network with strong control over national carbon flow. Interventions on these cross-community high-betweenness industrial bridges can produce larger overall carbon reduction effects than other types of strategic nodes.

(3) The network structure presents obvious community and small-world characteristics. The modularity index reaches 0.2089, far above the critical threshold, indicating a strong community structure. The overall distribution is a “pyramid” of one giant community, five medium communities, and multiple small/micro communities. Community 1 contains the most nodes (about 60% of the network), forming China’s core embodied carbon emission transfer community. Confirmed by comparison with equivalent random network baselines, the average clustering coefficient (0.6642) and average shortest path (1.9964) verify the typical small-world property—changes in a few key nodes can trigger structural responses of the entire network.

(4) Controllability analysis and ranking by degree, strength, and betweenness centrality identify 22 key controllable nodes and 4 isolated nodes. Among the three heuristic sorting schemes, nodes ranked by strength deliver the shortest total control transmission distance (57) and the best regulatory performance. Key control nodes are dominated by secondary and tertiary industries. From a topological and administrative perspective, this structural algorithm theoretically narrows down the targeted nodes by approximately 70% compared with indiscriminate, blanket emission reduction strategies. While this optimization significantly improves policy signal transmission paths, it is important to note that the absolute policy and economic equivalence of controlling these 26 nodes versus all 90 nodes has not been fully quantified. Future studies incorporating explicit economic abatement cost models are required to translate this structural optimization into concrete economic impact evaluations.

Finally, although this study has minimized the number of regulatory nodes and optimized policy transmission pathways through network control theory, it has not yet directly quantified the specific economic costs of implementing carbon control policies in particular provinces or industries. Future research should further integrate bottom-up engineering, technical, and economic data or marginal abatement cost (MAC) curves to comprehensively assess the actual economic costs of implementing optimal control strategies, thereby providing more precise policy guidance for carbon governance mechanisms.

### 5.2. Policy Recommendations

Combined with China’s dual-carbon goals and regional development layout, targeted suggestions for collaborative carbon abatement are put forward:

(1) Build a shared responsibility mechanism for cross-regional carbon transfer. As shown in the results, Jiangxi, Anhui, and Shandong are major net embodied carbon export provinces, while Jiangsu, Beijing, and Guangdong are major net import provinces. Led by relevant national authorities, net carbon importer regions could explore cooperative mechanisms with net export provinces—such as paired provincial cooperation for new energy development and traditional industrial green upgrading—to realize technology–resource complementary low-carbon development. The specific design of financial arrangements and institutional mechanisms is beyond the scope of this study and warrants further policy research.

(2) Carry out differentiated governance by community classification. Community 1, the giant community containing approximately 60% of all nodes and covering secondary and tertiary industries across nearly all provinces, should prioritize optimizing green supply chain access rules and establishing carbon monitoring mechanisms given its broad coverage and high internal connectivity. The five medium communities (Communities 2–6), primarily composed of primary industry nodes from specific provinces (e.g., JS1, AH1, SD1 in Community 2; ZJ1, JX1, SC1 in Community 3), need accelerated clean energy substitution tailored to agricultural and resource-based production. The 19 micro communities, each consisting of a single isolated primary industry node (e.g., BJ1, HeB1, IM1, SH1), are guided to embed into surrounding core industrial chains to realize low-carbon upgrading.

(3) Launch refined carbon regulation centering on key controllable nodes. The 22 key control nodes identified through strength ranking (Table 6)—including AH2, GD2, HeN2, ZJ3, JX2, SD3, and others—are predominantly secondary and tertiary industries and should serve as primary regulatory objects. Among these, nodes such as GD2 and HeN2 rank highly across multiple centrality indicators (Table 3), suggesting their particular strategic importance. The 4 isolated nodes (BJ1, HeB1, IM1, SH1) require supplementary direct supervision due to their structural disconnection from the main network. Together, these form a coordinated governance system covering approximately 30% of all nodes while achieving full network controllability.

(4) Link with existing institutional frameworks. The control nodes identified in this study could be integrated into carbon market quota incentives, preferential bank credit, and local government performance appraisal systems to strengthen policy implementation. How these institutional frameworks can be operationalized for the specific provincial-industrial nodes identified in this study warrants further investigation.

Project administration, Yixin Bao, Wenxia Chen and Chenhao Qian.

## Figures and Tables

**Table 1:** Top provinces by gross and net embodied carbon emission transfers in 2020 (Unit: billion tons).

Category	Rank 1	Rank 2	Rank 3
Gross Export	AH	JX	SD
(Total Outflow)	15.9599	12.1244	10.7571
Gross Import	AH	SD	JX
(Total Inflow)	12.5663	9.2065	8.4363
Net Export	JX	AH	SD
(Net Outflow)	3.6882	3.3935	1.5506
Net Import	JS	BJ	SC
(Net Inflow)	2.6775	1.3334	1.2385

**Table 2:** Topological and carbon flow indicators of three major industries in 2020. Definitions: Degree represents the number of connected nodes in the network; In-degree and out-degree are the number of direct import and export relationships, respectively; Strength (in-strength, out-strength) is the total weighted carbon flow volume after network threshold filtering, reflecting the actual effective carbon transmission scale; Import, export, and net export represent the total embodied carbon transfer volume before threshold filtering, covering all original transfer relationships; Betweenness centrality reflects the node’s ability to control carbon transmission paths in the network. Unit: billion tons. The large difference between total import/export (before threshold) and strength (after threshold) arises because strength only retains the top 14% high-weight edges after filtering, while import/export use the full unfiltered network.

Industry	Import	Export	Net Export	Degree	In-Degree
	(Billion Tons)	(Billion Tons)	(Billion Tons)		
primary	1.177	2.817	1.640	519	48
secondary	4.416	2.617	−1.799	1278	791
tertiary	3.383	3.542	0.159	1049	584
**Industry**	**Out-Degree**	**Strength**	**In-Strength**	**Out-Strength**	**Betweenness**
		**(Billion Tons)**	**(Billion Tons)**	**(Billion Tons)**	**Centrality**
primary	471	1.678	0.051	1.627	309.66
secondary	487	3.512	2.637	0.875	2849.81
tertiary	465	2.262	1.037	1.224	935.53

**Table 3:** Top 10 rankings by provincial-industry in-degree, out-degree, input intensity, output intensity, and betweenness centrality.

Number	In-Degree	Out-Degree	In Strength	Out Strength	Betweenness Centrality
1	JS2	JX1	AH2	JX1	GD2/589.19
2	GD2	AH1	JX2	AH1	HeN2/490.34
3	SC2	ZJ3	HeN2	HuB1	AH2/318.36
4	HeN2	HeN1	SD2	SD3	GS2/282.24
5	FJ2	AH2	HuB2	ZJ3	ZJ3/276.28
6	ZJ2	SD3	FJ2	AH2	FJ2/230.19
7	HuN2	HuB1	ZJ2	HeN1	HuB2/169.48
8	CQ2	JX3	JS2	AH3	HeN1/150.97
9	SD2	AH3	GD2	JX3	NX2/142.50
10	HuB2	GX3	HuN2	GX3	SD3/141.85

Note: (1) Province abbreviations: JS = Jiangsu; JX = Jiangxi; HeN = Henan; HuB = Hubei; GD = Guangdong; SD = Shandong; ZJ = Zhejiang; etc. (2) Node suffixes: 1 = Primary industry; 2 = Secondary industry; 3 = Tertiary industry.

**Table 4:** Group classification and sequence of some nodes.

Internal Node Sequence of Major Associations	
Club 1 (giant) (56 nodes)	BJ2, BJ3, TJ2, TJ3, HeB2, HeB3, SX2, SX3, IM2, IM3, LN1, LN2, LN3, JL2, JL3, HL2, HL3, SH2, SH3, JS2, JS3, ZJ2, ZJ3, AH2, AH3, FJ2, FJ3, JX2, JX3, SD2, SD3, HeN2, HeN3, HuB2, HuB3, HuN2, HuN3, GD2, GD3, GX2, HiN2, HiN3, CQ2, CQ3, SC2, SC3, GZ2, GZ3, YN2, YN3, SaX2, SaX3, GS2, GS3, XJ2, XJ3
Club 2 (medium)	JS1, AH1, SD1
Club 3 (medium)	ZJ1, JX1, SC1
Club 4 (medium)	HL1, HeN1, YN1
Club 5 (medium)	QH1, QH2, QH3
Club 6 (medium)	NX1, NX2, NX3
Small/Micro Communities (16 clubs, 19 nodes)	BJ1, TJ1 + HeB1, SX1, IM1, JL1, SH1, FJ1 + HuB1, GD1, GX1 + GX3, HiN1, CQ1, GZ1, HuB1, SaX1, GS1, XJ1
The basis for dividing the clubs	Micro communities (1 node), Small communities (2 nodes), Medium communities (3–5 nodes), Large communities (5–10 nodes), and Giant communities (over 10 nodes).

Note: The table shows that the 90 provincial-industrial nodes are partitioned into 26 communities in total: 1 giant community (56 nodes), 5 medium communities (15 nodes), 3 small communities, and 13 micro communities (19 nodes).

**Table 5:** Comparative analysis of different control strategies and control node sequences.

Sorting Criteria	Degree	Strength	Betweenness Centrality
Control Number of Nodes	26	26	26
Control Transmission Distance	61	57	60

**Table 6:** Classification of key control nodes and isolated nodes in the network.

Node Type	Number of Nodes	Node Sequence
Key control nodes	22	AH2, JX1, JX2, SD3, ZJ3, AH1, HeN2, HuB2, SD2, HuB1, FJ2, AH3, GD2, HuN2, HeN1, ZJ2, JX3, JS2, GX3, HeN3, GD3, GX2
Isolated nodes	4	BJ1, HeB1, IM1, SH1
Total	26	—

Note: Node abbreviations: 1 = Primary industry, 2 = Secondary industry, 3 = Tertiary industry. The 22 key control nodes are strictly selected based on strength ranking. The 4 isolated nodes (BJ1, HeB1, IM1, SH1) are primary industries with no connections to other nodes and must be directly controlled to achieve full network controllability.

## Data Availability

All the data used in this study are derived from the following publicly available authoritative statistical materials and databases: The China Carbon Emission Database (CEADs) https://www.ceads.net.cn/ (accessed on 20 May 2026).

## Appendix A

**Table A1:** Province Number and Abbreviation.

Number	Province	Abbreviation	Number	Province	Abbreviation
1	Beijing	BJ	16	Henan	HeN
2	Tianjin	TJ	17	Hubei	HuB
3	Hebei	HeB	18	Hunan	HuN
4	Shanxi	SX	19	Guangdong	GD
5	Inner Mongolia	IM	20	Guangxi	GX
6	Liaoning	LN	21	Hainan	HiN
7	Jilin	JL	22	Chongqing	CQ
8	Heilongjiang	HL	23	Sichuan	SC
9	Shanghai	SH	24	Guizhou	GZ
10	Jiangsu	JS	25	Yunnan	YN
11	Zhejiang	ZJ	26	Shaanxi	SaX
12	Anhui	AH	27	Gansu	GS
13	Fujian	FJ	28	Qinghai	QH
14	Jiangxi	JX	29	Ningxia	NX
15	Shandong	SD	30	Xinjiang	XJ

**Table A2:** Department—Industry Comparison Table.

Industry	Sector	Industry	Sector
1	Agriculture, Forestry, Animal Husbandry, Fishery Products and Services	2	Other Manufactured Products
2	Coal Mining and Dressing Products	3	Waste and Scrap
2	Petroleum and Natural Gas Extraction Products	3	Repair Services of Metal Products, Machinery and Equipment
2	Metal Ore Mining and Dressing Products	2	Production and Supply of Electric Power and Heat Power
2	Non-metal and Other Ore Mining Products	2	Production and Supply of Gas
2	Food and Tobacco	2	Production and Supply of Water
2	Textiles	2	Construction
2	Textile Wearing Apparel, Footwear, Leather, Down and Related Products	3	Wholesale and Retail Trade
2	Wood Processing Products and Furniture	3	Transportation, Warehousing and Postal Services
2	Paper Making, Printing, Cultural, Educational and Sporting Goods	3	Accommodation and Catering Services
2	Petroleum, Coking Products and Nuclear Fuel Processing Products	3	Information Transmission, Software, and Information Technology Services
2	Chemical Products	3	Finance
2	Non-metallic Mineral Products	3	Real Estate
2	Metal Smelting and Rolling Processing Products	3	Leasing and Business Services
2	Metal Products	3	Scientific Research and Technical Services
2	General Equipment	3	Management of Water Conservancy, Environment and Public Facilities
2	Special Purpose Equipment	3	Resident Services, Repair and Other Services
2	Transportation Equipment	3	Education
2	Electrical Machinery and Equipment	3	Health and Social Work
2	Communication Equipment, Computers and Other Electronic Equipment	3	Culture, Sports and Entertainment
2	Instruments and Meters	3	Public Administration, Social Security and Social Organizations
